# Chemistry of Bridged Lactams: Recent Developments

**DOI:** 10.3390/molecules24020274

**Published:** 2019-01-12

**Authors:** Roman Szostak, Michal Szostak

**Affiliations:** 1 Department of Chemistry, Wroclaw University, F. Joliot-Curie 14, 50-383 Wroclaw, Poland; roman.szostak@chem.uni.wroc.pl; 2 Department of Chemistry, Rutgers University, 73 Warren Street, Newark, NJ 07102, USA

**Keywords:** amide bond, bridged lactams, twisted amides, amides, Winkler-Dunitz parameters, N–C activation, hypersensitivity, nitrogen heterocycles, distortion, bridged sultams

## Abstract

Bridged lactams represent the most effective and wide-ranging method of constraining the amide bond in a non-planar conformation. A previous comprehensive review on this topic was published in 2013 (*Chem. Rev.* **2013**, *113*, 5701–5765). In the present review, which is published as a part of the *Special Issue on Amide Bond Activation*, we present an overview of the recent developments in the field of bridged lactams that have taken place in the last five years and present a critical assessment of the current status of bridged lactams in synthetic and physical organic chemistry. This review covers the period from 2014 until the end of 2018 and is intended as an update to *Chem. Rev.* **2013**, *113*, 5701–5765. In addition to bridged lactams, the review covers recent advances in the chemistry of bridged sultams, bridged enamines and related non-planar structures.

## 1. Introduction

**Figure 1:**
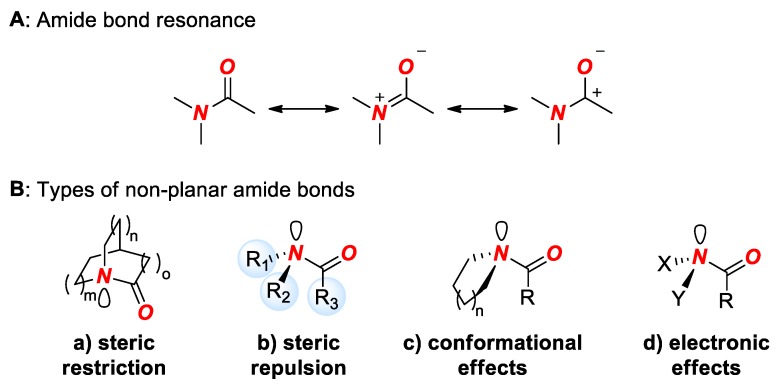
(**A**) Amide Bond Resonance. (**B**) Types of Distorted Amide Bonds.

The amide bond is arguably the most important linkage in chemistry and biology [1]. Typical amide bonds are planar as a result of amidic resonance (n_N_ → π*_C=O_ conjugation, 15–20 kcal/mol) (Figure 1A) [2].

**Figure 2:**
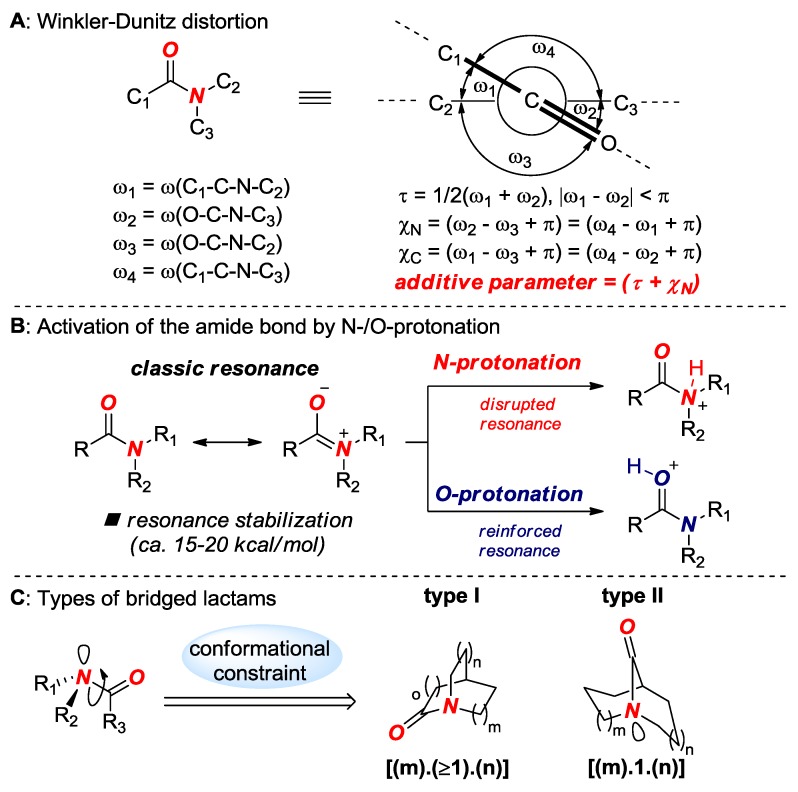
(**A**) Winkler-Dunitz Distortion. (**B**) Activation of the Amide Bond by *N*-/*O*-Protonation. (**C**) Types of Bridged Lactams.

The redesign of the amide bond geometry through structural and electronic changes of substituents comprising the amide bond has had a profound impact on the physico-chemical properties of amides [3,4,5,6]. The alteration of the amide bond geometry generally leads to a reversal of traditional properties of amides, such as lower barrier to cis-trans rotation, increased length of the N-C(O) bond, favored protonation at the nitrogen atom, and increased reactivity in nucleophilic addition and hydrolysis [3,4,5,6]. The geometric and structural changes of the amide bond are an established technique to affect properties of amide bonds in biology and medicinal chemistry [7,8,9,10], while recent advances in selective metal insertion into the amide bond driven by its distortion represent a thriving and general concept in organic synthesis [11,12]. In general, amide bond distortion can be achieved by four methods (Figure 1B): (1) steric restriction, (2) steric repulsion, (3) conformation effects, and (4) electronic effects. Out of these methods, the most effective one by far is steric restriction. Typically, steric restriction involves constraining the amide bond in a rigid bicyclic ring system with a nitrogen atom positioned at a bridgehead position. This allows one to constrain the typical planar amide bond in a non-planar conformation with the magnitude of distortion principally controlled by the type of ring system (Figure 2). To date, bridged lactams represent the only method that has allowed for a substantial distortion, exceeding 60% of the maximum theoretical value of the amide bond [3,4,5,6,11,12].

Amide bond distortion is measured by Winkler-Dunitz parameters: τ (twist angle), χ_Ν_ (pyramidalization at N) and χ_C_ (pyramidalization at C) [13] as well as by changes in N–C(O) and C=O bond lengths (Figure 2A). Amide bond distortion leads to a change of thermodynamic *N*-/*O*-protonation aptitude, which is a key effect that controls the reactivity of non-planar amide bonds (Figure 2B) [11]. The properties of amide bonds in bridged lactams are further amplified by a type of bridged lactam scaffold (Figure 2C). In general, bridged lactams are classified into amides in which the N–C(O) bond is placed on a one-carbon bridge or on a larger bridge, with the former enjoying additional stabilization through transannular scaffolding effects. 

In this review, published as a part of the *Special Issue on Amide Bond Activation*, we present an overview of the recent developments in the field of bridged lactams and present a critical assessment of the current status of bridged lactams. This review covers the period from 2014 until the end of 2018 and is intended as an update to the previous comprehensive review on topic, *Chem. Rev.* **2013**, *113*, 5701–5765 [3]. In addition to bridged lactams, the review covers recent advances in the chemistry of bridged sultams, bridged enamines and related non-planar structures. For additional coverage, the reader is referred to previous reviews on bridged lactams [4,5,6]. It is our hope that the review will serve as a useful reference for chemists involved in various aspects of activating the amide bond and stimulate further research in this area.

## 2. Synthesis, Properties and Reactivity of Bridged Lactams

Recent advances in the field of bridged lactams include: (1) identification of the additive Winkler-Dunitz parameter, (2) synthesis of extremely twisted non-stabilized amides, (3) synthesis of novel bridged lactams, and (4) new examples of reactivity of non-planar amides. 

**Figure 3:**
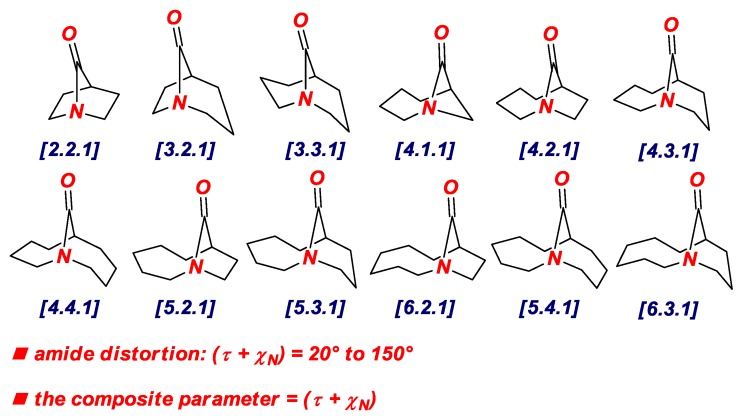
Additive Winkler-Dunitz Distortion Parameter: Sum of Twist Angle and Nitrogen Pyramidalization (Στ + χ_N_).

**Figure 4:**
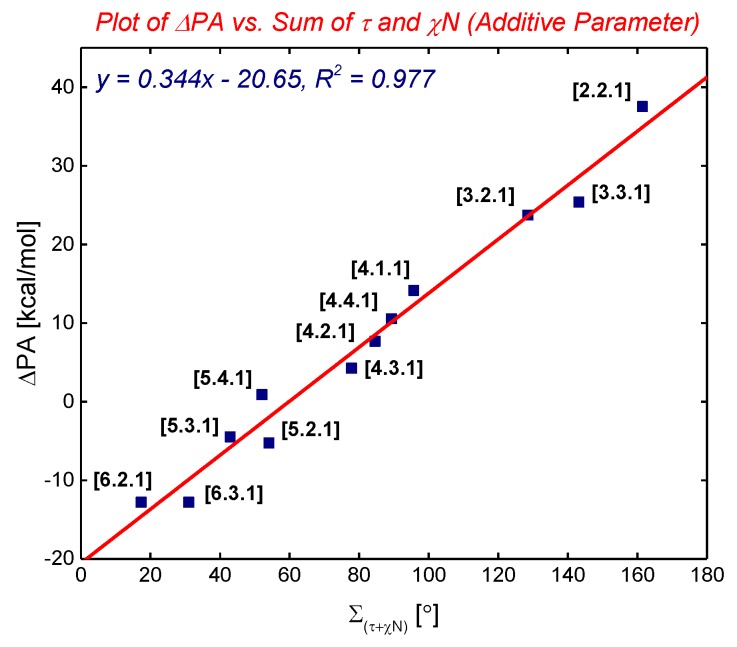
Plot of ΔPA (PA = Experimental Protonation Affinity) to the Sum of Twist and Pyramidalization at Nitrogen Angles (Additive Winkler-Dunitz Parameter: Στ + χ_N_). Note that (Στ + χ_N_) gives linear correlations vs. structural and other energetic parameters. See [15]. Also note comprehensive studies [16,17,18].

In 2015, we have identified the additive Winkler-Dunitz distortion parameter (Στ + χ_N_), sum of twist and pyramidalization at nitrogen angles, as a more accurate prediction of the structural and energetic properties of non-planar amides than either twist or pyramidalization alone (Figure 3 and Figure 4) [14,15]. A computational study to determine the effect of amide distortion on *N*-/*O*-protonation using a set of lactams comprehensively covering the entire distortion range (Figure 3) revealed a linear correlation between the composite Winkler-Dunitz parameter (Στ + χ_N_) and *N*-/*O*-protonation aptitude (Figure 4) [14]. Our subsequent study demonstrated that the additive Winkler-Dunitz parameter (Στ + χ_N_) gives linear correlations vs. structural and other energetic parameters (resonance energies, atomic charges, frontier molecular orbitals, infrared frequencies) [15]. 

**Figure 5:**
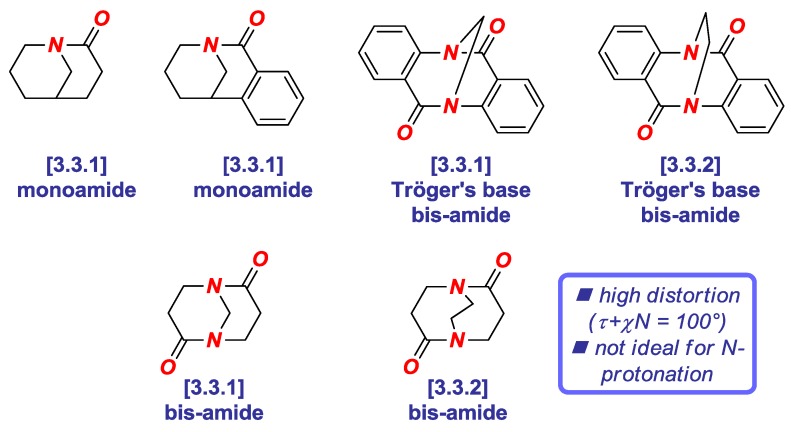
Additive Winkler-Dunitz Distortion Parameter in Tröger’s Base Twisted Amides.

Since (1) amide bond distortion typically hinges upon both twist and pyramidalization, and (2) the additive Winkler-Dunitz parameter gives a more accurate prediction of geometric changes of the amide bond, this parameter should be routinely reported to describe structural variations of all non-planar amide bonds. Recently, we have utilized the additive Winkler-Dunitz parameter (Στ + χ_N_) to determine the origin of high twist and *N*-/*O*-protonation aptitude of Tröger’s base twisted amides (Figure 5) [19]. Perhaps surprisingly, we found that although Tröger’s base twisted bis-amides are among the most twisted amides synthesized and structurally-characterized to date (*vide infra*), these amides are less effective in probing *N*-protonation than less twisted in this series 1-azabicyclo [3.3.1] nonan-2-one derivatives.

**Figure 6:**
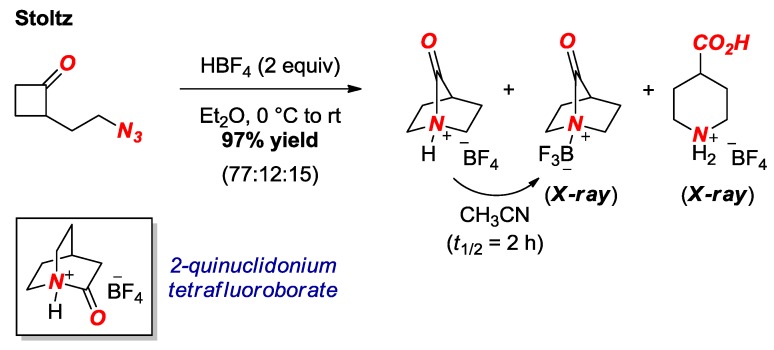
Synthesis of 7-Hypoquinuclidonium Tetrafluoroborate.

In 2016, the extremely twisted 7-hypoquinuclidonium tetrafluoroborate was reported by Stoltz and co-workers (Figure 6) [20]. The group has impressively exploited the intramolecular Aubé-Schmidt reaction to access the [2.2.1] bridged scaffold. This unconventional amide bond forming strategy represents a general approach to this and another extremely twisted amide, 2-quinuclidonium tetrafluoroborate (see Figure 6, box) [21], in the absence of nucleophiles that would likely decompose both compounds. It should be noted that the target twisted amide was isolated as a HBF_4_ salt or BF_3_ complex. The latter compound was fully characterized by X-ray crystallography, revealing one of the most twisted amide bonds isolated to date (τ = 90.0°, χ_N_ = 69.8°; N–C(O) = 1.526 Å, C=O = 1.186 Å).

It is now well-established that *N*-coordination of the amide bond increases twist and pyramidalization (*vide infra*). Thus, with the exception of a structurally-unique 1-adamantan-2-one derivatives (see below), there are no examples of unconstrained structurally-characterized amides with a combined Winkler-Dunitz parameter (Στ + χ_N_) exceeding 100° in the neutral form.

**Figure 7:**
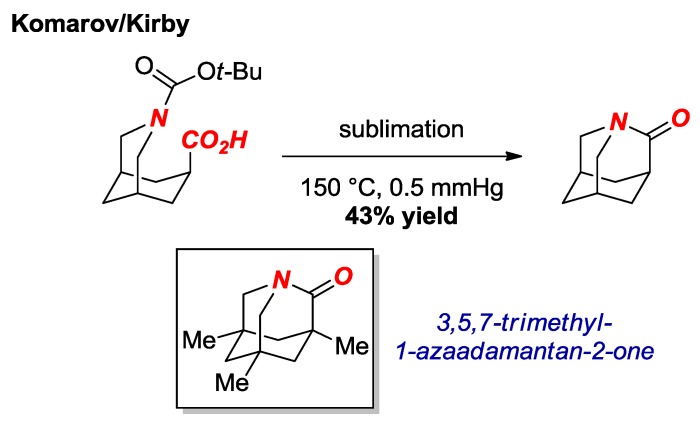
Synthesis of Parent 1-Azaadamantan-2-one.

In 2016, the synthesis of another extremely twisted amide was reported by Komarov, Kirby et al. (Figure 7) [22]. These researchers achieved the synthesis of the parent 1-aza-2-adamantanone via a route consisting of thermal amidation of the *N*-Boc protected amino acid. Previous calculations showed a significant stabilizing effect of the methyl groups in the trimethyl twisted amide derivative (see Figure 7, box) [23]. The parent 1-aza-2-adamantanone was fully characterized after protonation as HBF_4_ salt (τ = 88.1°, χ_N_ = 58.0°; N-C = 1.508 Å, C=O = 1.186 Å). Furthermore, the authors obtained the X-ray structure of the α-monomethylated 1-aza-2-adamantanone in a neutral form (τ = 90.0°, χ_N_ = 61.8°; N-C = 1.448 Å, C=O = 1.201 Å). Similar to the [2.2.1] amide synthesized by the Stoltz group (cf. [2.2.2] amide), the parent 1-aza-2-adamantanone was found to be more reactive in reactions with nucleophiles than the previous “most twisted amide” 3,5,7-trimethyl-1-azaadamantan-2-one [24].

**Figure 8:**
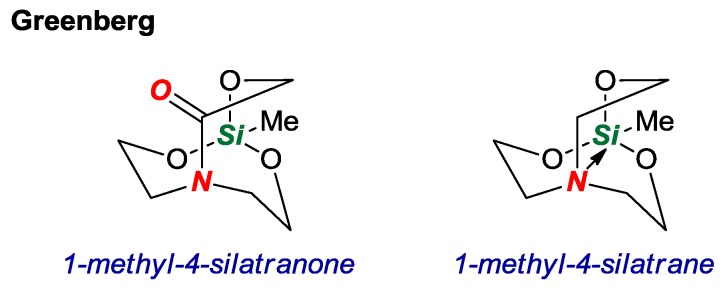
Computational Study of 1-Methyl-4-Silatranone.

In 2017, Greenberg and co-workers reported an interesting study of a silicon-containing twisted amide in a [3.3.3] scaffold (Figure 8) [25]. Heteroatom-containing derivatives of bridged lactams have received considerable attention as a means of facilitating the synthesis and tuning properties of the twisted amide bond. Through computations, the authors demonstrated that the nitrogen atom in 1-methyl-4-silatranone would be more similar to a lactam rather than a silatrane with a long intramolecular N-Si bond (N-Si = 2.902 Å vs. 1-methylsilatrane, N-Si = 2.466 Å). Although the attempted synthesis via condensation of (HOCH_2_CH_2_)_2_N(COCH_2_OH) with various silanes was unsuccessful, the study lays a foundation for the synthesis of silicon-containing twisted amides.

**Figure 9:**
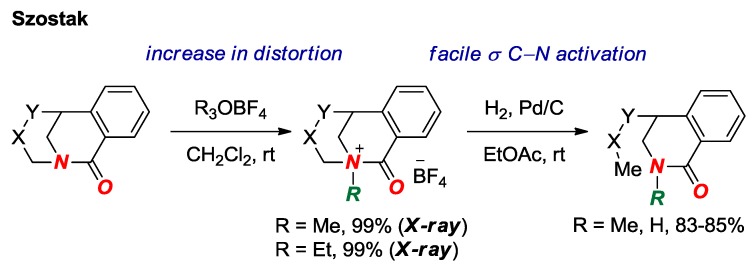
*N*-Alkylation of Bridged Lactams as a Trigger for σ N-C Bond Activation.

In 2016, we reported the first examples of structurally-characterized *N*-alkylated bridged lactams (Figure 9) [26]. *N*-Alkylation significantly increased amide bond distortion (τ = 44.0°, χ_N_ = 58.3°; N-C = 1.554 Å, C=O = 1.192 Å). Furthermore, we demonstrated that *N*-coordination activated the twisted amide bond towards σ N–C bond activation by Pd-catalyzed hydrogenation.

**Figure 10:**
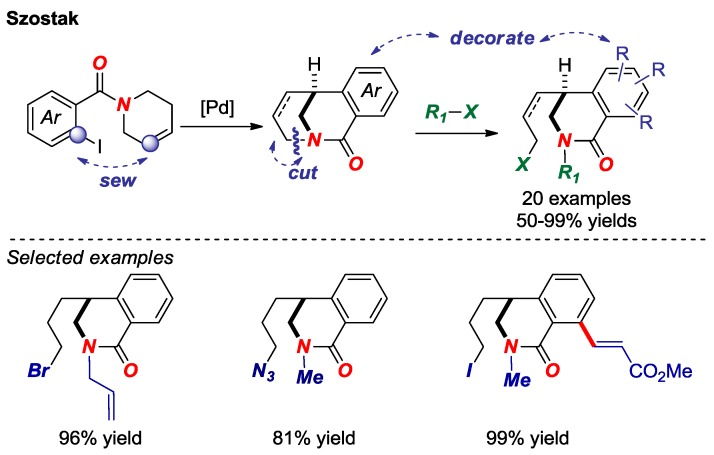
Sew-And-Cut of Bridged Lactams by a Transition-Metal-Free σ N–C Bond Activation.

Transition-metal-free σ N-C bond activation in bridged lactams was reported by our group in 2017 (Figure 10) [27]. Facile assembly of the twisted amide scaffold by intramolecular Heck reaction, followed by *N*-alkylation and selective σ bond cleavage established a “sew-and-cut” approach to complex isoquinoline-2-ones by a formal di-functionalization of the N-C amide bond. The reactivity was correlated with amide bond twist in that less distorted amides were found unreactive. Given the utility of mild difunctionalization methods in organic synthesis, twisted lactams are attractive intermediates for the synthesis of nitrogen-containing heterocycles by this approach.

**Figure 11:**
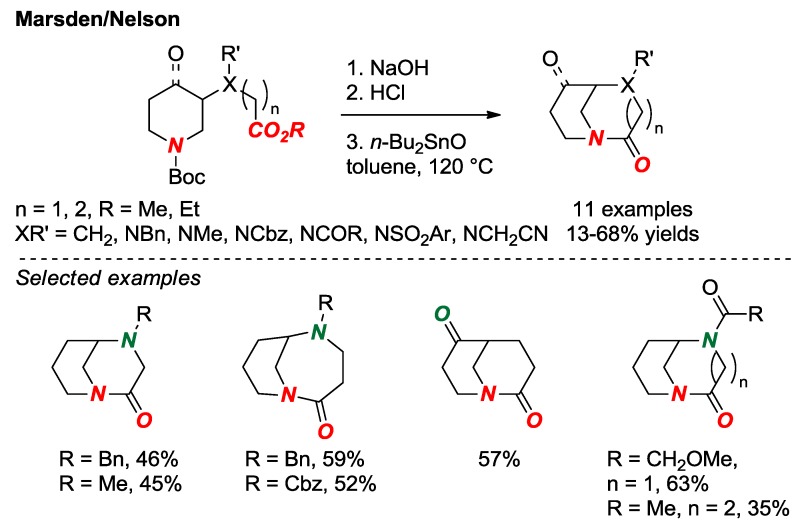
Fragment-Based Drug Discovery using Bridged Lactams.

In 2018, Marsden, Nelson and co-workers reported the synthesis of a set of bridged lactams with [3.3.1] and [4.3.1] scaffolds as part of their research on fragment-based drug discovery (Figure 11) [28]. This approach nicely utilizes the presence of an additional heteroatom in the twisted amide structure to facilitate the synthesis of starting materials. The cyclization was carried out according to the established lactamization protocol mediated by Bu_2_SnO [29]. Due to the unique shape diversity, bridged lactams hold a significant potential as unexplored scaffolds in drug discovery.

**Figure 12:**
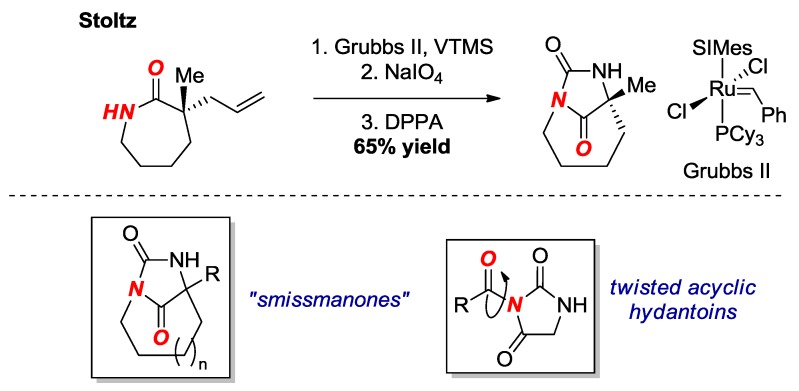
Synthesis of a Bridged Hydantoin. VTMS = Vinyloxytrimethylsilane. DPPA = diphenyl phosphoryl azide. SIMes = 1,3-Bis(2,4,6-trimethylphenyl)-2-imidazolidinylidene.

In 2017, Stoltz and co-workers reported the synthesis of a bridged hydantoin by asymmetric α-allylation, Ru-catalyzed olefin isomerization, oxidative cleavage, Curtius rearrangement and *N*-cyclization onto the isocyanate (Figure 12) [30]. The cyclization is performed by the same mechanism as reported previously by Brouillette [31]; however, the method avoids the use of toxic lead acetate. The bridged hydantoin was fully characterized by X-ray crystallography (τ = 36.4°, χ_N_ = 50.2°; N-C(O) = 1.404 Å; C=O = 1.210 Å). This research illustrates one of the few methods for the synthesis of an enantioenriched twisted amide bond.

Bridged hydantoins of this type were originally proposed by Smissman as potential anticonvulsants (Figure 12, box) [32]. Our group has recently reported the structural characterization of related acyclic twisted *N*-acyl-hydantoins (Figure 12, box) [33].

**Figure 13:**
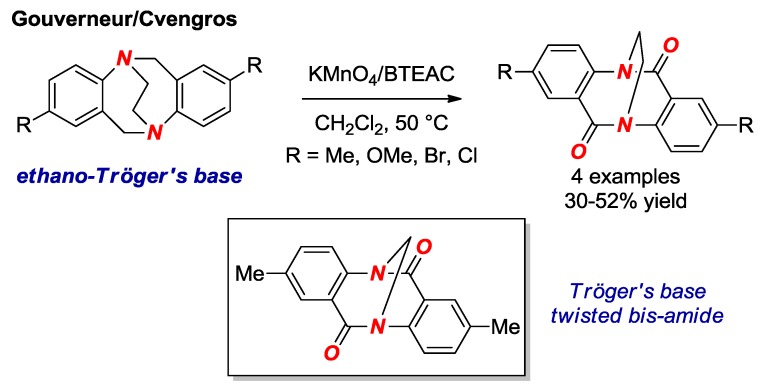
Synthesis of Ethano Tröger’s Base Twisted Amides. BTEAC = Benzyltriethylammonium Chloride.

In 2017, Gouverneur, Cvengros and co-workers reported the synthesis of ethano Tröger’s base twisted bis-amides (Figure 13) [34]. Their approach involves one-step oxidation of the ethano-Tröger’s base precursors under the conditions reported earlier by Wärnmark (Figure 13, box) [35]. The ethano Tröger’s base twisted bis-amide is significantly twisted (τ = 29.8°, χ_N_ = 45.5°; N–C(O) = 1.401 Å, C=O = 1.215 Å). The advantage of this method is rapid access to a twisted amide bond; however, our study suggests that the presence of the fused aromatic ring is detrimental to the *N*-protonation reactivity in this class of twisted amides [19].

**Figure 14:**
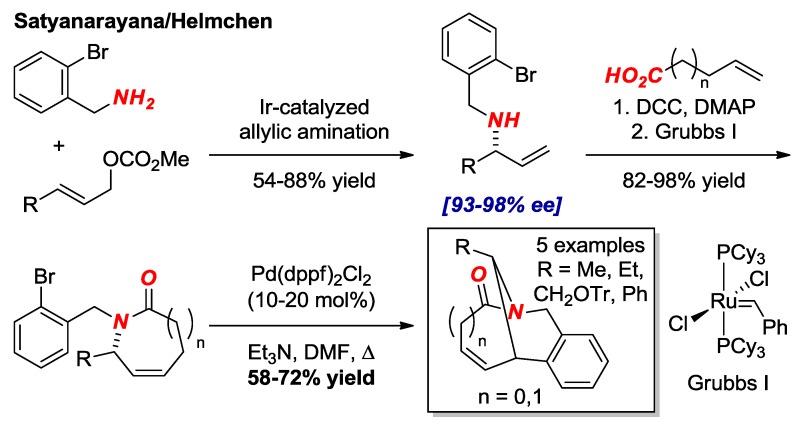
Enantioselective Synthesis of Bridged Lactams by Allylic Amination.

Satyanarayana and Helmchen reported asymmetric synthesis of bridged lactams in [3.3.1] and [4.3.1] scaffolds using Ir-catalyzed allylic amination as an enantioselectivity determining key step (Figure 14) [36]. With allylic amines in hand, the synthesis was completed by well-established amidation/Heck cyclization sequence. The structure of one of the lactams in the [3.3.1] series was confirmed by X-ray crystallography (τ = 30.8°, χ_N_ = 52.7°; N–C(O) = 1.393 Å, C=O = 1.218 Å).

**Figure 15:**
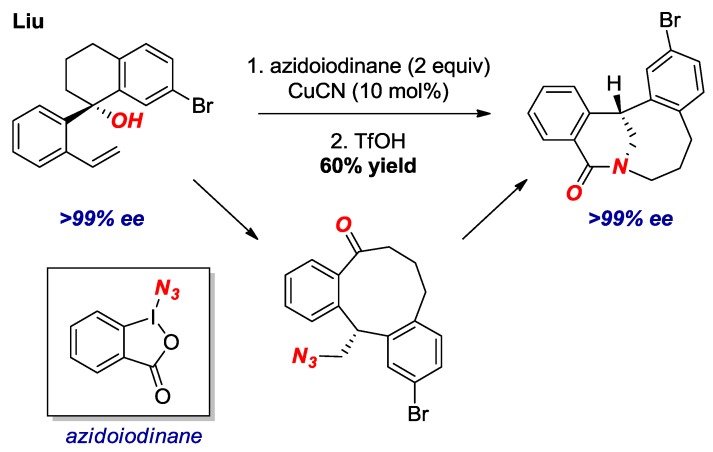
Enantioselective Synthesis of Bridged Lactams by Radical Aryl Migration.

An inventive strategy for the synthesis of bridged lactams in the [5.3.1] and larger scaffolds was reported by Liu and co-workers (Figure 15) [37]. The authors developed a new process for radical aryl migration with chirality transfer to form macrocyclic ketones. As an application of this method, they subjected several azido-ketones to the transannular Aubé-Schmidt rearrangement, resulting in the formation of bridged lactams in 40–63% yields. Nevertheless, bridged lactams with the overall sum of carbon atoms forming the bridged structure of ten or more, are similar in properties to planar amides.

## 3. Bridged Sultams

Bridged sultams (bridged sulfonamides) have attracted significant attention due to a wide range of biological activities of the sulfonamide bond [38]. In contrast to bridged lactams, constraining a sulfonamide bond in rigid bicyclic ring systems is easily possible due to the lack of Nlp to SO_2_ conjugation (Nlp = nitrogen lone pair) [39]. Such bridged sultams are not hyper-reactive to hydrolysis and besides applications in medicinal chemistry have been used as template in stereoselective synthesis enabled by rapid scission of the N-SO_2_ bond [3].

**Figure 16:**
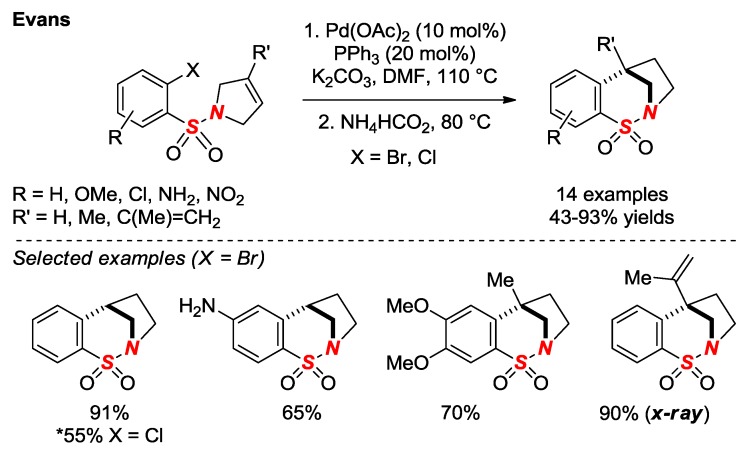
Synthesis of Saturated Bridged Sultams via Intramolecular Heck Reaction/Reduction.

In 2017, Evans and co-workers in the continuation of their studies on bridged sultams reported an improved method for the synthesis of saturated sultams via intramolecular reductive Heck reaction (Figure 16) [40]. The use of a single Pd catalyst and a broad substrate scope are noteworthy features of this method. One of the saturated sultams in a [3.2.1] scaffold was characterized by X-ray crystallography (*θ* = 328.7; N-S = 1.643). The same group reported a bromonium-triggered 1,2-Wagner-Meerwein rearrangement of benzofused bridged sultams (not shown) [41].

**Figure 17:**
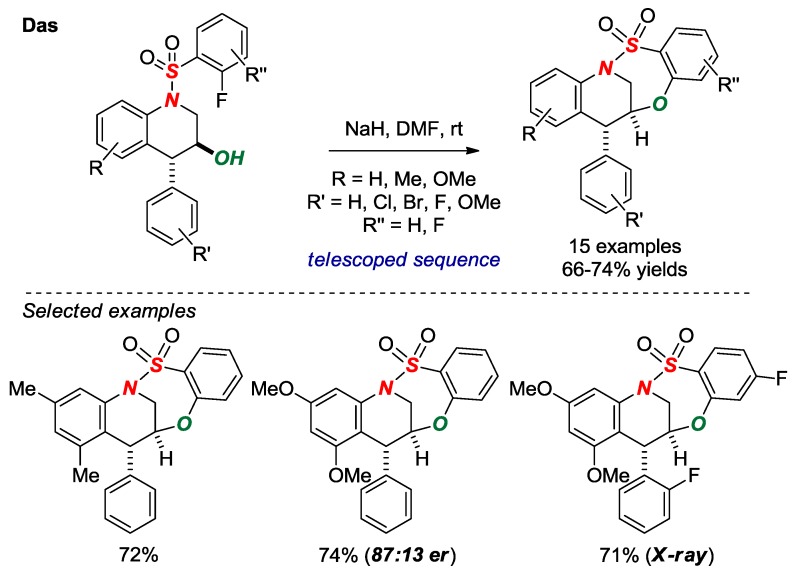
Synthesis of Bridged Benzothiaoxazepine-1,1-dioxides via S_N_Ar Cyclization.

In 2017, Das and co-workers nicely exploited intramolecular S_N_Ar-type cyclization to form bridged benzothiaoxazepine-1,1-dioxides in a [4.3.1] scaffold (Figure 17) [42]. An advantage of this method is a rapid, telescoped, three-step synthesis of bridged sultams from the corresponding *N*-aryl-2-fluorobenzenesulfonamides and trans-2,3-epoxy cinnamyl tosylates. One of the sultams was fully characterized by X-ray crystallography (*θ* = 342.0; N-S = 1.668). The authors have also developed an enantioselective variant by using a chiral trans-2,3-epoxy cinnamyl alcohol.

**Figure 18:**
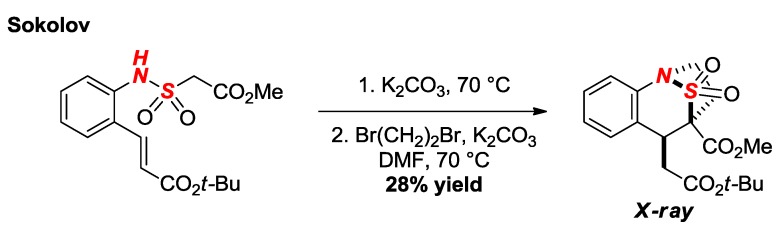
Synthesis of an Apex Bridged Sultam via 1,4-Addition.

One example of an “apex-type” bridged sultam in a [3.2.1] scaffold was reported by Sokolov and co-workers (Figure 18) [43]. The X-ray structure demonstrated significant pyramidalization of the nitrogen atom (*θ* = 325.5; N–S = 1.668).

## 4. Application in Natural Product Synthesis

Bridged lactams continue to serve as useful intermediates in the total synthesis of natural products [3]. In general, recent applications hinge upon the increased electrophilicity of the carbonyl group and increased nucleophilicity of the nitrogen atom of the amide bond rendered possible by geometric distortion.

**Figure 19:**
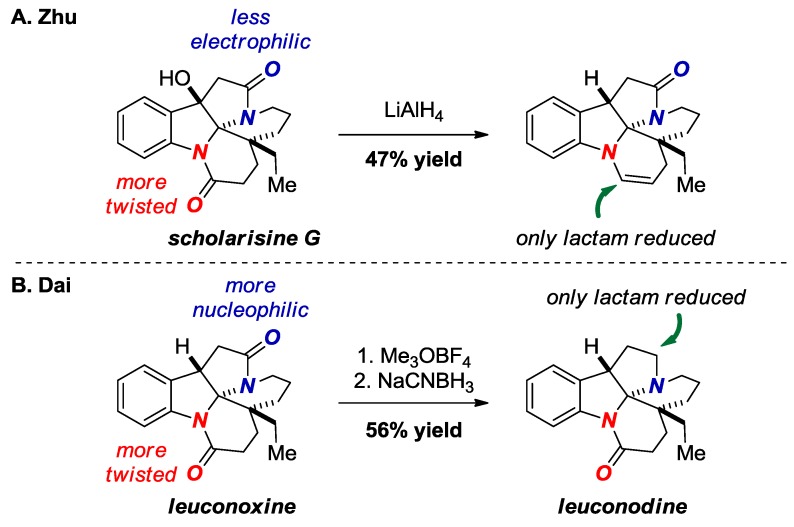
Reduction of a Bridged Lactam in Scholarisine G (**A**) and Leuconoxine Alkaloids (**B**).

In 2015, Zhu and co-workers reported selective reduction of the more twisted amide bond in scholarisine G (Figure 19A) [44]. The enamine was obtained after dehydration of the intermediate hemiaminal. The X-ray structure of scholarisine G showed a significantly distorted *N*-aryl amide bond (τ = 21.9°, χ_N_ = 32.0°; N-C(O) = 1.373 Å, C=O = 1.223 Å) vs. the aliphatic amide bond (τ = 2.9°, χ_N_ = 19.9°; N-C(O) = 1.352 Å, C=O = 1.227 Å). It is also possible that the selective reduction in this case can be explained by the exclusive stability of the five-membered ring lactams and by the fact that the six-membered lactam is an anilide. In contrast, Dai exploited the higher basicity of the oxygen atom in the aliphatic amide bond in a structurally-related leuconoxine to selectively maneuver reduction of the more electron-rich amide bond through electrophilic pathway (Figure 19B) [45].

**Figure 20:**
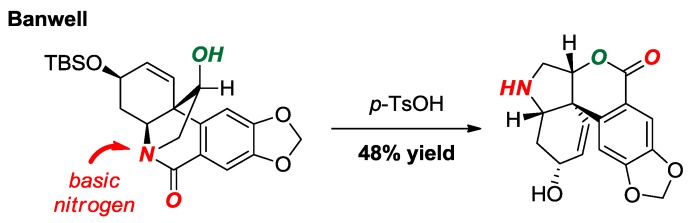
Lactam-to-Lactone Rearrangement of a Bridged Lactam in Haemanthidine Alkaloids.

A total synthesis of 3-*O*-demethylmacronine, an Amaryllidaceae alkaloid, utilizing a lactam-to-lactone rearrangement of a twisted amide was reported by Banwell and co-workers (Figure 20) [46]. This elegant method capitalized on the high basicity of the twisted amide nitrogen atom to form the acylium ion, which underwent trapping with a pendant hydroxyl group under mild conditions.

**Figure 21:**
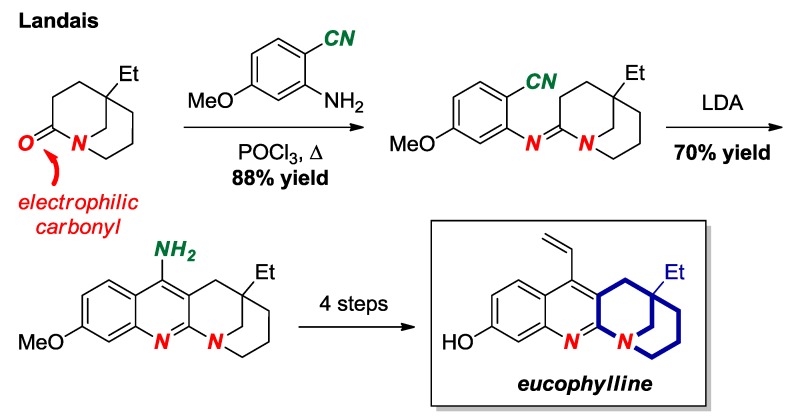
Bridged Amidine from a [3.3.1] Bridged Lactam in the Synthesis of Eucophylline.

Landais and co-workers reported a total synthesis of eucophylline, a dimeric terpene indole alkaloid, taking advantage of a high electrophilicity of the carbonyl group in a [3.3.1] bridged lactam scaffold (Figure 21) [47]. Bridged lactams are known to readily condense with amines to form amidines. In this approach, condensation of the twisted amide bond with aniline afforded a bridged amidine, which provided the key disconnection to the eucophylline core.

## 5. Miscellaneous Examples

**Figure 22:**
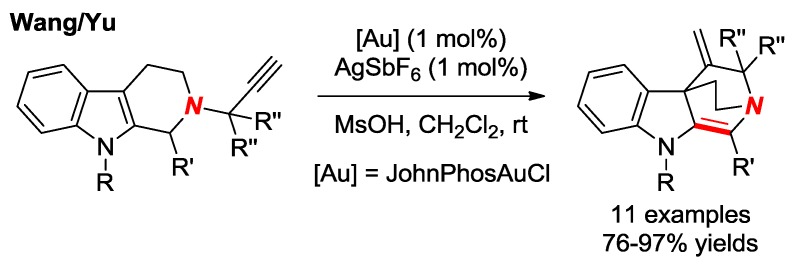
Synthesis of Bridged Enamines via Gold-Catalyzed Spriocyclization.

In 2015, Wang, Yu and co-workers reported the synthesis of bridged enamines via Au-catalyzed spiro-cyclization of 2-propargyl-β-tetrahydrocarbolines (Figure 22) [48]. In these heterocycles, the resonance interaction between Nlp and π electrons of the double bond is inhibited, resulting in a nucleophilic nitrogen atom.

**Figure 23:**
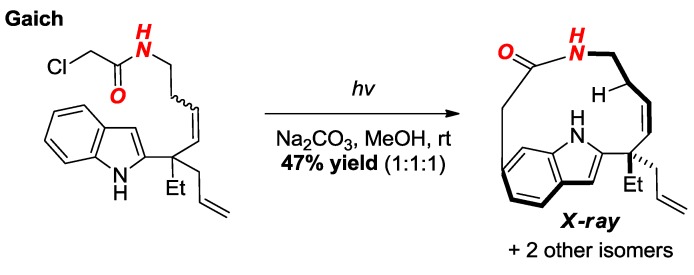
Synthesis of a Macrocyclic Lactam via Witkop Cyclization.

In their studies on the Witkop cyclization, Gaich and co-workers reported the synthesis of macrocyclic amides supported by the indole ring (Figure 23) [49]. The structure of one of the amides was confirmed by X-ray crystallography and showed a significantly distorted amide bond (τ = 32.3°, χ_N_ = 0.0°; N–C(O) = 1.353 Å, C=O = 1.227 Å).

**Figure 24:**
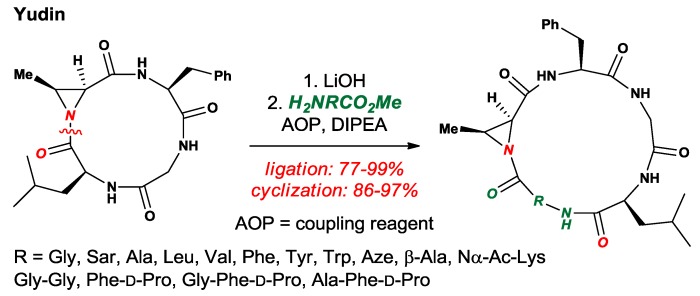
Cyclic Twisted Amide-Containing Tetrapeptides.

Yudin and co-workers developed several elegant methods for site-specific incorporation of amino acids [50], peptide sequencing [51] and conformational control [52] of cyclic peptides based on the twisted amide electrophilic sites (Figure 24). In their approach, the integration of a highly strained and *N*-pyramidalized aziridinyl ring allows for selective N–C(O) cleavage and amino acid incorporation, while the strained aziridinyl ring provides a handle for further functionalization by aziridine-ring opening with nucleophiles.

Several additional studies on non-planar amides have been reported. Computational studies on ion-pair interactions in Kirby’s amide HBF_4_ salts [53] and *N*-protonation of twisted amides by HF and HCl [54] were reported by Panday. Mykhailiuk and co-workers reported selective σ N-C bond scission in strained acyclic amides [55]. Stereoselective C-O bond cleavage in pyramidalized diketopiperazines induced by amide bond distortion was reported by Jahn and co-workers [56]. Intramolecular hydrogen bonding to electron-deficient oxygen in *N*-pyramidalized bicyclic amides was reported by Otani, Ohwada and co-workers [57]. Excellent reviews on the role of amide bond activation in biological molecules [58], amidicity [59], and heteroatom substitution at amide nitrogen [60] have been published. Additional studies on the properties of amides have been reported [61,62]. Recent relevant studies on acyclic non-planar amides should also be noted [11,12,63,64,65,66].

## 6. Conclusions

In summary, we have reviewed recent advances in the area of bridged lactams. This field continues to provide the most effective and wide-ranging method to achieve non-planarity of the amide bond. The main progress in the last five years includes: (1) identification of the additive Winkler-Dunitz parameter as a straightforward and accurate descriptor of the structural and energetic properties of the non-planar amide bond, and (2) the synthesis of extremely twisted bridged lactams in both quinuclidone and adamantanone series. Recently reported examples of novel bridged lactam scaffolds, divergent N–C cleavage reactivity and applications in the total synthesis of natural products are also worth noting.

Despite the considerable progress that has been made, the area is far from being mature. The recent remarkable progress in the chemistry of acyclic twisted amides has underlined the benefits of using conformational restriction (i.e., bridged lactams) to achieve non-planarity, but also brought to light the deficiencies of bridged lactams, and non-planar amide bonds in general. Most important is that except for the structurally-limited 1-aza-2-adamantanones there is a complete lack of isolated and fully characterized unhindered bridged lactams with a combined Winkler-Dunitz parameter (Στ + χ_N_) exceeding 100° in the neutral form (or in other words merely 60% of the maximum theoretical distortion). It is beyond belief that the area has progressed so little since the first conception of a bridged lactam bond by Lukeš in 1938.

Further, very little is known about the stability of bridged lactams and, by extension, of non-planar amides. It is now safe to assume that a range of distortion of (Στ + χ_N_) ca. 70–80° gives isolable lactams. However, this stability range has only been tested in a very limited set of lactams.

Even less is known about the generality of amide bond distortion, properties and structures across different sets of non-planar amides. This research is hindered by the very few types of non-planar bridged lactams that have been described to date. The recent example of Tröger’s base twisted amides is as a good case in point [19]. Despite significant amide bond distortion, these lactams do not protonate readily as a consequence of Nlp conjugation onto the aromatic ring.

Another issue involves the reactivity of non-planar amides. Almost all research thus far has focused on simple hydrolytic stability and nucleophilic addition studies. At present, only one type of potentially very synthetically appealing metal-catalyzed σ N-C bond activation has been reported, and the mechanism of this transformation is unknown.

The importance of the amide bond and the continuous role of twisted amides in chemistry and biology have served as a powerful stimulus for fundamental research on bridged lactams. Given the obvious possibilities for applications of non-planar amide bonds, future studies are expected to have a broad impact beyond this important research area.
